# Influence of sire fertility status on conceptus-induced transcriptomic response of the bovine endometrium

**DOI:** 10.3389/fcell.2022.950443

**Published:** 2022-08-22

**Authors:** E. O’Callaghan, J.M. Sánchez, M.B. Rabaglino, M. McDonald, H. Liu, T.E. Spencer, S. Fair, D.A. Kenny, P. Lonergan

**Affiliations:** ^1^ School of Agriculture and Food Science, University College Dublin, Dublin, Ireland; ^2^ Spanish National Research Council, National Institute for Agricultural and Food Research and Technology (CSIC-INIA), Madrid, Spain; ^3^ Division of Animal Sciences, University of Missouri, Columbia, MO, United States; ^4^ Laboratory of Animal Reproduction, School of Natural Sciences, Biomaterials Research Cluster, Bernal Institute, Faculty of Science and Engineering. University of Limerick, Limerick, Ireland; ^5^ Teagasc Animal and Grassland Research and Innovation Centre, Dunsany, Ireland

**Keywords:** bull fertility, pregnancy establishment, uterine receptivity, embryo development, gene expression

## Abstract

The aim was to examine the effect of sire fertility status on conceptus-induced changes in the bovine endometrial transcriptome. To generate elongated conceptuses, Day 7 blastocysts produced *in vitro* using frozen-thawed sperm from Holstein Friesian bulls (3 High fertility, HF and 3 Low fertility, LF) were transferred in groups of 5–10 into synchronized heifers (*n* = 7 heifers per bull) and recovered following slaughter on Day 15. Day 15 endometrial explants recovered from the uterine horn ipsilateral to the corpus luteum were recovered from synchronized cyclic heifers (*n* = 4). Explants from each heifer were co-cultured for 6 h in RPMI medium alone (Control) or with 100 ng/ml ovine recombinant interferon tau (IFNT) or with a single conceptus from each HF or LF bull. After 6 h, explants were snap frozen and stored at −80°C. Extracted mRNA was subjected to RNA-seq and the resulting data were analyzed with R software. The numbers of differentially expressed genes (DEG; FDR<0.05) were: HF vs. Control: 956; LF vs. Control: 1021; IFNT vs. Control: 1301; HF vs. LF: 2. Unsurprisingly, the majority of DEG (658) were common to all comparisons and were related to IFNT-induced changes in the endometrium. Prior to applying the adjusted *p*-value, there were 700 DEG between HF and LF, with 191 and 509 genes more expressed in HF or LF, respectively (*p* < 0.05). Overrepresentation analysis of KEGG pathways (FDR<0.05), revealed that DEG with higher expression in LF were involved in cell cycle and proteolysis, while those upregulated DEG by HF conceptuses were strongly associated with immune process pathways, such as TNF, NF-kappa B, cytokine-cytokine receptor interaction, and TLR signaling. These pathways were also enriched by DEG upregulated by IFNT compared to the Control. Furthermore, only the HF, and not the LF group, affected the expression of most genes in these pathways (*p* < 0.05) according to a negative binomial regression model. Finally, a weighted gene co-expression network analysis revealed two clusters of co-expressed genes associated with the HF conceptuses (*p* < 0.05), which were also enriched for the aforementioned pathways. In conclusion, HF conceptuses, similar to IFNT treatment, stimulated multiple pathways involved in immune response, which were apparently not affected by LF conceptuses.

## 1 Introduction

Pregnancy loss is recognized as a major cause of reproductive failure in cattle. While fertilization success is typically high following artificial insemination (AI), many of the resulting embryos fail to develop to term. A significant proportion of pregnancy loss occurs between fertilization and maternal recognition of pregnancy (Diskin and Morris, 2008; Berg et al., 2010; Pohler et al., 2020) which in cattle occurs around Day 16 post fertilization. In high-producing dairy cows, as many as 50% of embryos may be non-viable by Day 7 (Sartori et al., 2010; Wiltbank et al., 2016). A further proportion of loss occurs in the period of conceptus elongation and interferon-tau (IFNT) production associated with signalling to the uterus leading to failure of maternal recognition of pregnancy (Wiltbank et al., 2016; Forde and Lonergan, 2017). While it is generally accepted that post fertilization maternal factors dominate in regulating pregnancy establishment, the contribution of the sire to early embryo loss is less well characterized (Daigneault, 2021).

In Bos indicus beef cows, while no difference in pregnancy rate was observed at Day 30 among sires used for timed AI, pregnancy loss during the second month of gestation was highly variable among sires (1.4–11.1%) (Franco et al., 2018). Similarly, Bos taurus beef cows exhibited significant variation in pregnancy loss between Days 24 and 31 of gestation (1.8–11.7%) and between Days 31 and 60 of gestation (2.3–12.6%) among sires used for timed AI (Franco et al., 2020). The incidence of pregnancy loss was associated with lower circulating pregnancy-associated glycoprotein concentrations on Days 28 and 30 of gestation which may be reflective of compromised embryo/conceptus development (Pohler et al., 2016; Franco et al., 2018).

While the direct contribution of the sire to embryo loss is unclear, the reduced ability of embryos from low fertility (LF) bulls to establish pregnancy is likely multifactorial and associated with sperm fertilizing ability, early embryo development, and events after conceptus elongation (Kropp et al., 2017; Ortega et al., 2018; Gross et al., 2019). A recent study from our group suggests an influence of the sire on early embryo development and early conceptus survival (O'Callaghan et al., 2021); while differences in sire fertility were not reflected in fertilization rate, differences in embryo quality were apparent as early as Day 7 and likely contributed to a higher proportion of conceptuses sired by high fertility (HF) bulls surviving to Day 15 (O'Callaghan et al., 2021). In the context of AI, where one bull can sire thousands of pregnancies, any underlying subfertility issues could have a major cumulative impact on pregnancy rate, reproductive wastage and subsequent production efficiency. This is important when one considers that despite the fact that such bulls have passed rigorous laboratory sperm quality assessments, they can vary by more than 20% points in their field fertility (Fair and Lonergan, 2018).

Optimal dialog between the developing embryo and endometrium during the peri-implantation period is essential for pregnancy recognition and uterine receptivity in preparation for implantation and placentation (Hue et al., 2012; Brooks et al., 2014). The absolute requirement for interaction between the developing embryo and the maternal endometrium is progressively acquired as early pregnancy progresses. Up to the blastocyst stage, no contact is necessary, as evidenced by the ability to produce embryos *in vitro* using IVF and the success of transferring such embryos to a virgin uterus on Day 7. Indeed, early studies elucidating the day of maternal recognition of pregnancy, involving the transfer of embryos at progressively later times, demonstrated that it was possible to establish pregnancy by embryo transfer as late as Day 16 (Betteridge et al., 1980). Post-hatching development, however, during which the bovine blastocyst undergoes a period of morphological transformation, progressing from a spherical to a tubular and then filamentous structure, is a uterine-driven process and has not been recapitulated *in vitro*. Furthermore, on the maternal side, conceptus-derived IFNT is required to prevent the mechanisms that induce luteolysis. In this context, it has been demonstrated that the endometrium can act as a “sensor” of embryo quality with its transcriptome being reflective of the developmental competency of the conceptus present (Sandra et al., 2015). For example, the transcriptomic response of the uterus to the conceptus differs between those derived from AI vs. somatic cell nuclear transfer (Bauersachs et al., 2009; Mansouri-Attia et al., 2009), those derived from the transfer of *in vivo* vs. *in vitro* produced blastocysts (Mathew et al., 2019) and age-matched short and long conceptuses (Sánchez et al., 2019). While the majority of these differences are associated with IFNT, some of the altered genes are apparently induced by conceptus-derived, but IFNT-independent, factors (Bauersachs et al., 2009; Mathew et al., 2019; Sánchez et al., 2019).

With this background, the aim of this study was to examine the effect of sire fertility status on conceptus-induced changes in the endometrial transcriptome which could potentially explain differences in fertility between sires used in AI. We hypothesised that bovine endometrial tissue exposed to conceptuses derived from high compared with low fertility bulls would exhibit a different transcriptional profile consistent with greater potential for successful pregnancy establishment.

## 2 Materials and methods

All experimental procedures involving animals were sanctioned by the Animal Research Ethics Committee of University College Dublin and were licensed by the Health Products Regulatory Authority, Ireland, in accordance with Statutory Instrument No. 543 of 2012 under Directive 2010/63/EU on the Protection of Animals used for Scientific Purposes.

### 2.1 Bulls

Frozen-thawed semen from 3 HF to 3 LF Holstein-Friesian bulls was used; these were the same bulls as used in our previous study mentioned above (O'Callaghan et al., 2021). All bulls had passed the standard quality control tests in the AI centre before release into the field. Data on field fertility were obtained from the Irish Cattle Breeding Federation (ICBF) database based on an adjusted sire fertility index (Berry et al., 2011). Sire fertility was defined as pregnancy rate to a given service identified retrospectively either from a calving event or where a repeat service or a pregnancy scan deemed the animal not to be pregnant. These raw data were then adjusted for factors including semen type (frozen, fresh), cow parity, month of service, day of the week when serviced, service number, cow genotype, herd, AI technician and bull breed. The adjusted sire fertility index given for each bull was then weighted for the number of service records, resulting in an adjusted pregnancy rate (mean = 0%). Holstein-Friesian bulls with a minimum of 500 inseminations formed the base population (840 bulls), from which HF (*n* = 3; +4.37%) and LF (*n* = 3; −12.7%) bulls were selected with an average difference based on adjusted fertility scores of 17.1% (O'Callaghan et al., 2021).

### 2.2 Experimental animals and procedures

**FIGURE 1:**
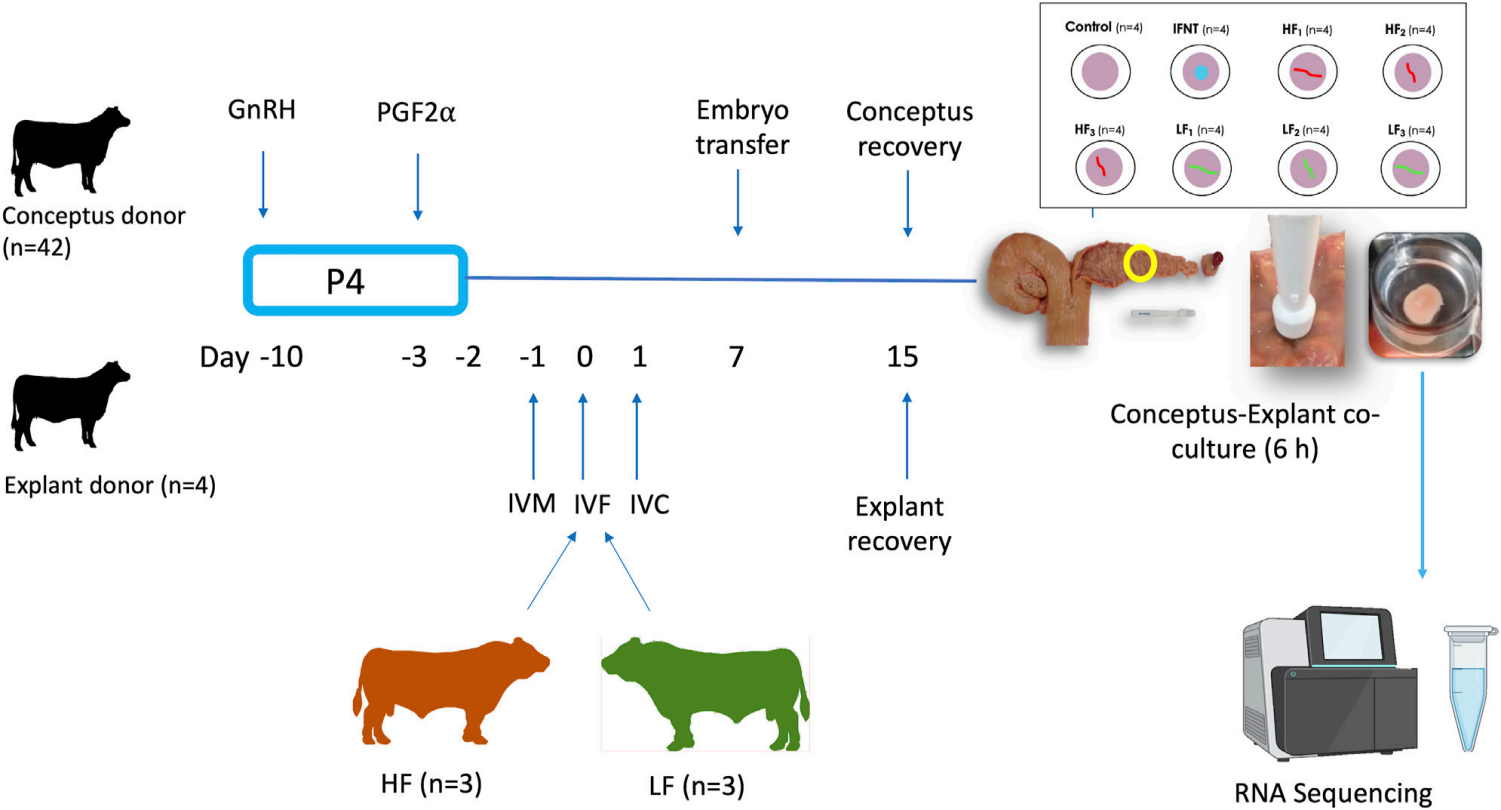
Experimental design. Estrous cycles of conceptus and explant donors were synchronized using an 8-days progesterone (P4) releasing device with GnRH at PRID insertion and prostaglandin 2α the day before device removal. In parallel, oocytes recovered from slaughterhouse ovaries were matured *in vitro* (IVM), fertilised (IVF) with semen from these 6 bulls and cultured *in vitro* (IVC) to generate blastocysts for transfer. The day of IVF was the equivalent to the day of ovulation in the heifers. Day 7 blastocysts from each high fertility (HF) and low fertility (LF) bull were transferred in groups of 5–10 per bull into each conceptus donor, with 7 heifers allocated per bull, in order to generate elongated conceptuses. All heifers were slaughtered on Day 15. Following recovery, single conceptuses of similar length were co-incubated with endometrial explants for 6 h. Eight endometrial explants were recovered from each of the 4 explant donors. Explants from each explant donor were exposed to medium alone (Control; 4 explants), 100 ng/ml ovine recombinant interferon tau (IFNT; 4 explants), or a single Day 15 conceptus from each of the HF bulls (HF_1-3_; 12 explants) and LF (LF_1-3_; 12 explants) bulls. After the 6 h co-culture, explants were processed for RNA sequencing.

The experimental design is illustrated in Figure 1. Charolais- or Limousin-cross beef heifers (*n* = 46, 24.3 months ±4.7, 608 kg ± 27.9) were used as conceptus donors (*n* = 42) or explant donors (*n* = 4). In order to generate elongated conceptuses, Day 7 *in vitro* produced blastocysts (*n* = 5–10 per heifer) from one of the six bulls were transferred into each conceptus donor (7 heifers per bull). Heifers designated as explant donors did not receive embryo transfer. All animals were maintained under identical conditions during the study and were fed a diet consisting of grass and maize silage supplemented with a standard beef ration. Estrous cycles of all heifers were synchronized using an 8-days progesterone releasing intravaginal device (PRID E, 1.55 g of progesterone, Ceva Santé Animale). On the day of the PRID E insertion each heifer was administered 2-mL of a synthetic GnRH (Ovarelin, Ceva Santé Animale, equivalent to 100 μg of Gonadorelin) intramuscularly. One day before PRID E removal all heifers were administered 5 mL of PGF2α (Enzaprost, Ceva Santé Animale, equivalent to 25 mg of Dinoprost) intramuscularly to induce luteolysis of the corpus luteum (CL). Only those heifers observed in standing estrus (Day -1) were used. Simultaneously on Day -1, *in vitro* oocyte maturation for production of blastocysts commenced for transfer on Day 7. All animals underwent transrectal ovarian ultrasonography using a portable ultrasound machine (Easi-Scan; BCF Technology Ltd., Bellshill, Scotland, United Kingdom) fitted with a 4.5–8.5-MHz linear array transducer and on Day 7 of the estrous cycle immediately before embryo transfer (ET) to recipients to assess CL location. On Day 7, *in vitro*-derived embryos (*n* = 5 to 10; Day 7, Code 1–2 according to the manual of the International Embryo Technology Society) were pooled before being loaded into straws for transcervical transfer to the uterine horn ipsilateral to the ovary bearing the CL. All heifers (conceptus donors and explant donors) were slaughtered in a commercial abattoir on Day 15 (8 days-post ET).

### 2.3 *In vitro* embryo production

Blastocysts were produced *in vitro* using methods described previously (Rizos et al., 2002). Briefly, immature cumulus–oocyte complexes (COCs) were recovered by aspirating follicles from the ovaries of heifers and cows slaughtered at a local abattoir, washed in PBS, and matured for 24 h in groups of 50 in 500 μL TCM-199 supplemented with 10% fetal calf serum and 10 ng/mL epidermal growth factor at 39°C under an atmosphere of 5% CO_2_ in air with maximum humidity. Matured COCs were inseminated with frozen-thawed sperm from one of the six bulls at a concentration of 1 × 10^6^ sperm/mL. Gametes were co-incubated for 20 h at 39°C in an atmosphere of 5% CO_2_ in air with maximum humidity. Presumptive zygotes were denuded by gentle vortexing and cultured in synthetic oviduct fluid droplets (25 μL; 25 embryos per droplet) at 39°C in a humidified atmosphere with 5% CO_2_ and 5% O_2_ under mineral oil. Blastocysts for transfer to recipient heifers were removed from culture, pooled per bull, and loaded into straws (5–10 embryos per straw) on Day 7 (IVF = Day 0).

### 2.4 Endometrial explant procedure

Immediately after collection, the reproductive tract of each explant donor (n = 4) was placed on ice and transported to the laboratory within approximately 2 h. Once in the laboratory, the ipsilateral uterine horn was dissected from the remainder of the tract, washed with 1% phosphate buffered saline (PBS; Gibco) containing (v/v) 1% 100 X antibiotic-antimycotic (ABAM; Gibco), and sprayed with 70% ethanol. Next, endometrium from the intercaruncular areas of the middle third of the horn was isolated as described previously (Borges et al., 2012). Briefly, the endometrium was exposed by opening the uterine horn longitudinally along the antimesometrial side, and the uterine luminal surface was washed with 1% PBS containing 1% ABAM. Then, an 8 mm biopsy punch was used to obtain a portion of the intercaruncular endometrium and myometrium. Sterile scissors were then used to completely remove the myometrium from the intercaruncular endometrium biopsy. After collection, the endometrial explants (50–80 mg) were washed in conical tubes containing 25 mL of Hank’s balanced salt solution (HBSS; Gibco) containing 1% ABAM. The media was poured off, and explants were washed a further two times in 25 mL of HBSS without ABAM before placing them upright individually in culture wells (4-well plate with 15 mm diameter x 11 mm deep) containing 1 mL of Roswell Park Memorial Institute (RPMI) medium (Gibco) medium plus 1% ABAM.

Culture wells with endometrial explants placed epithelial side up were incubated in 5% CO_2_ in air at 38.8°C for 4 h. The media was aspirated and replaced with 1 ml of pre-warmed fresh media (RPMI with 1% ABAM). Then, eight explants from each heifer were cultured individually in RPMI for 6 h in 5% CO_2_ in air at 38.8°C with either 1) nothing (Control; *n* = 4), 2) 100 ng/mL recombinant ovine IFNT (provided by G. Charpigny, INRAE, France; *n* = 4), or 3) a single Day 15 conceptus from one of each of the six bulls (HF: *n* = 12; LF: *n* = 12). In order to minimize variation, explants from the same uterus were used across all treatments in each of the four replicates. After incubation, conceptuses were removed from the top of the endometrial explant and the explants were individually snap-frozen in liquid nitrogen and stored at −80°C for RNA extraction.

### 2.5 Explant RNA extraction and RNA sequencing

A total of 32 endometrial explants (individual explant was the experimental unit in this study) were sequenced (8 from each of the 4 explant donors, as described above; Figure 1). Total RNA was isolated from frozen explant endometrium (∼40 mg) by homogenizing in 1 ml of TRIzol reagent and a Qiagen RNeasy Mini Kit (Qiagen) as described previously (Mathew et al., 2019). RNA quantity and quality were determined using an Agilent Bioanalyzer (Agilent Technology). To eliminate DNA contamination, samples were treated with Dnase. RNA concentrations and integrity were determined by quantitative high-sensitivity RNA analysis on the Fragment Analyzer instrument (Catalog # DNF-472, Advanced Analytical Technologies, Inc., Ankeny, IA). RNA library preparation and sequencing was conducted by the University of Missouri DNA Core facility as previously described (Moraes et al., 2018).

Paired-end sequencing was performed on an Illumina NextSeq 500 sequencer to a depth of 30–40 million raw reads per sample. FastQC (v 0.11.9) and MultiQC (v 1.10.1) were used to evaluate the quality of the sequence. Trimmomatic (v 1.10.1) was used to remove adapters and low-quality bases from the reads. Then they were aligned to the bovine genome (ARS-UCD1.2.103) by applying STAR aligner (v 2.7.0e) with an average unique mapping rate of 88.5% and a average read length of 201 bp. Gene quantification was calculated by using StringTie package (v 2.1.3b). Assembled transcripts for each sample were merged and created a uniform set of transcripts for all samples. Counts calculate for the samples is provided by the StringTie python script. Data are available at the Gene Expression Omnibus database (GSE205404).

### 2.6 Bioinformatics

**FIGURE 2:**
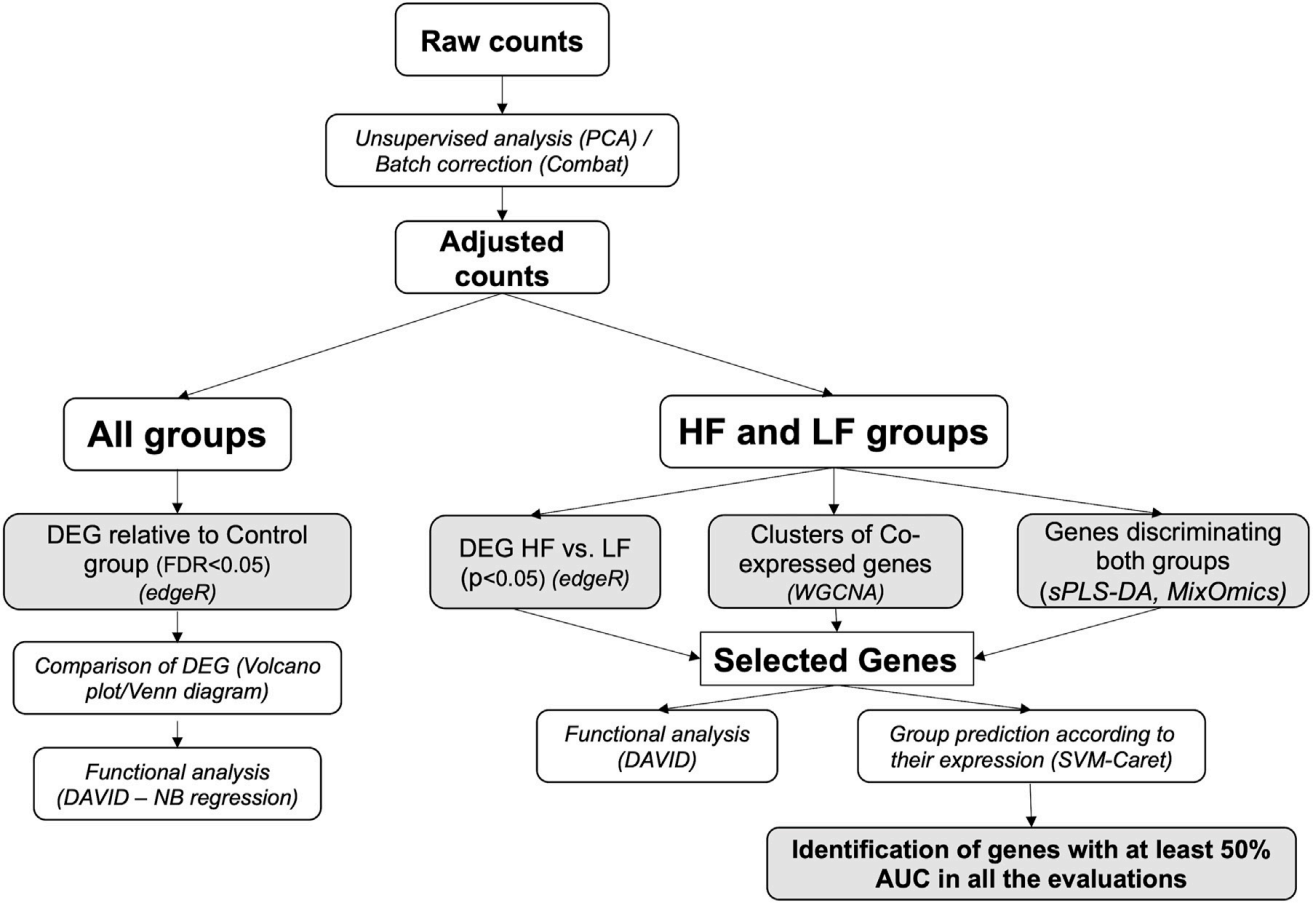
Bioinformatics pipeline. The scheme represents the workflow employed in the analysis of RNAseq data obtained from endometrial explants exposed to nothing (Control), interferon tau, or a Day 15 conceptus from a high fertility (HF) or low fertility (LF) bull. The methods are detailed in italics (the software used is in parenthesis). Shaded squares denote the main outputs from the study. PCA: principal component analysis; DEG: differentially expressed genes; WGCNA: weighted-gene co-expression network analysis; sPLS-DA: sparse partial least square-discriminant analysis; DAVID: Database for Annotation, Visualization and Integrated Discovery; AUC: area under the curve.

Data were analyzed through appropriate bioinformatic packages within the R software platform (R Core Team (2020)). An initial unsupervised analysis using a principal component analysis (PCA) plot of raw data to determine sample distribution based on transcriptome profile highlighted the effect of individual heifer (explant donor) on the endometrial transcriptome. Thus, data were adjusted using the CombatSeq function of the Surrogate Variable Analysis (SVA) package (Johnson et al., 2006) (Supplementary Figure S1). Next, four approaches were employed to analyze the adjusted data. Firstly, for all groups, differentially expressed genes (DEG) for each group relative to the Control group were determined and compared. However, as the differences in the endometrial response to embryos conceived by HF or LF bulls were subtle, three additional approaches were applied to the data from these two groups to discern and individualize the endometrial transcriptome in each case. These methods are detailed below and illustrated in Figure 2.

### 2.7 Determination of DEG and functional analysis for all groups

The edgeR package (Robinson et al., 2010) was used for filtering, normalization and DEG identification. Genes with less than one count per million in 4 or more samples (smaller class), were filtered out before normalization (Rau et al., 2013). Filtered data were normalized through weighted trimmed mean of M-values (Robinson and Oshlack, 2010). Next, data dispersion was estimated through a maximization of the negative binomial likelihood. Finally, a negative binomial generalized log-linear model was fit to read counts for each transcript and conduct genewise likelihood ratio tests for the coefficient contrast (McCarthy et al., 2012). The pairwise comparisons were: HF vs. Control, LF vs. Control, IFNT vs. Control and HF vs. LF. DEG were defined as those with false discovery rate (FDR) < 0.05. Resulting DEG in each contrast were visualized through volcano plots and compared in Venn diagrams to determine overlapping genes.

Functional analysis of the DEG was carried out using Database for Annotation, Visualization and Integrated Discovery (DAVID) (Dennis et al., 2003), to determine enriched KEGG pathways (FDR<0.05). Based on these results, some pathways were chosen to determine the effect of each group on the expression of all the genes in such pathways through a negative binomial regression model, using the MASS package for the R software (Venables and Ripley, 2002). The complete list of gene Entrez IDs belonging to the selected pathways was downloaded from the KEGG pathway database (https://www.genome.jp/kegg/pathway.html). If more than two Ensembl IDs were encoding for the same Entrez ID, the average read counts were averaged and rounded.

### 2.8 Identification of endometrial genes responding to conceptuses derived from HF or LF bulls

#### 2.8.1 Determination of DEG

This procedure was performed as explained above for all groups, although genes with less than one count per million in 12 or more samples were filtered out before normalization. DEG between HF versus LF groups were defined as those with a *p*-value <0.05 and were visualized in a volcano plot and a PCA plot. Functional analysis was performed with DAVID to determine enriched KEGG pathways (FDR <0.05) by DEG upregulated or downregulated by HF bulls.

#### 2.8.2 Weighted gene coexpression network analysis

This analysis was carried out with the Weighted gene coexpression network analysis (WGCNA) package (Langfelder and Horvath, 2008). Gene counts were filtered to retain 6254 transcripts, 50% of the most variable ones, and afterwards, values were normalized by variable stabilizing transformation (Huber et al., 2003). The automatic method was employed for block-wise signed network construction and module detection. The co-expression similarity was raised to a soft thresholding power (β) of 8 to calculate adjacency. The resulting modules were related with each group (LF or HF) to identify clusters of co-expressed genes significantly correlated with them, which were visualized through box plots. Genes in these clusters were subjected to functional analysis with DAVID to determine enriched KEGG pathways (FDR<0.05). Selected pathways were graphed with the Pathview package (Luo and Brouwer, 2013). Briefly, this tool set maps the data with the pathway of interest and creates the pathway graph. Genes in these pathways with more expression in HF or LF groups were colored in red or green, respectively.

#### 2.8.3 Supervised analysis: Sparse partial least square discriminant analysis

Selection of the most discriminative genes between HF and LF groups was conducted using Sparse partial least square discriminant analysis (sPLS-DA) (Le Cao et al., 2011), with the mixOmics package (Ka et al., 2009). Genes were filtered to retain those highly expressed genes, i.e., with more than 100 counts per million in 12 or more samples, and were transformed through variance stabilizing transformation (Huber et al., 2003). The algorithm was run with one component, as G-1 components could extract sufficient information to discriminate all phenotype groups, G being the number of groups. The maximum number of genes able to discriminate between both groups was determined by choosing increasing increments of 50 genes and evaluating the spread of the samples in the first component in a PCA score plot, and the standard deviation (SD) of the first component. The number of genes selected were those for which the first component showed a SD lower than 2. Selected genes were visualized through hierarchical clustering and a heat map, which depicts the gene expression level in each sample. The clustering was made using Spearman Rank Correlation as similarity metric and centroid linkage as clustering method, implemented with the Cluster 3.0 software (de Hoon et al., 2004). The resulting dendrogram and the heat map were visualized with Java TreeView (Saldanha, 2004).

### 2.9 Identification of the most important endometrial genes among those responding to conceptuses derived from HF or LF bulls

The expression of genes selected by the three methods detailed above was employed to predict the status (HF or LF) of each sample through a machine learning approach. One third of the 24 samples were randomly selected as testing set while the remaining samples constituted the training set. The method chosen was Support vector machines (Support vector classifier) with linear kernels (SVM), implemented with the kernlab package (Karatzoglou et al., 2004) through the caret package (Kuhn, 2008). Leave-one-out cross validation method was employed as the internal control for the training dataset. Next, genes were sorted according to their importance, i.e., to the area under the ROC curve (AUC) for each predictor (gene). This procedure was repeated 10 times for each of the three lists of genes. Genes which consistently showed at least 50% AUC for each list were retained and compared between the three lists in a Venn Diagram. Genes that were shared between at least two lists were visualized through a PCA plot and a dendrogram/heatmap.

## 3 Results

Data on conceptus recovery following ET have been previously reported (O'Callaghan et al., 2021). Briefly, overall conceptus recovery rate on Day 15 based on number of blastocysts transferred on Day 7 was 52.1% (185/355). The mean (±SEM) number of conceptuses recovered per recipient on Day 15 was 4.95 ± 0.57 for HF bulls, 3.85 ± 0.51 for LF bulls, and 4.40 ± 0.39 overall. Conceptus recovery rate was higher in HF (59.4%, 104/175) versus LF (45.0%, 81/180; *p* < 0.05). Overall, mean (±SEM) conceptus length was 24.4 ± 2.0 mm and was not affected by fertility status.

### 3.1 Endometrial genes differentially expressed between each group and the control

**FIGURE 3:**
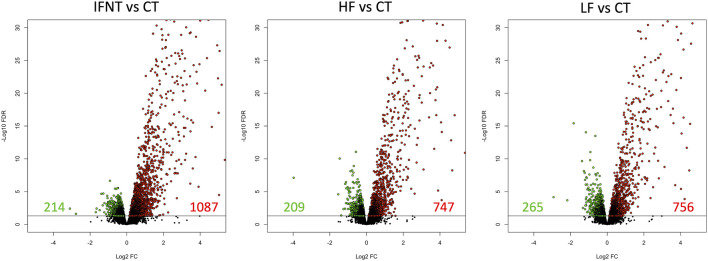
Volcano plots of the differentially expressed genes between each group and the Control. A volcano plot is a type of scatterplot that shows statistical significance on the Y-axis versus magnitude of change on the X-axis. Each spot represents a gene; those on the right (in red) are upregulated, while those on the left (in green) are downregulated. The remainder (in black) are not differentially expressed. The horizontal black line was drawn at FDR = 0.05. RNAseq data from endometrial explants exposed to nothing (Control, CT), interferon tau (IFNT), or a Day 15 conceptus from a high fertility (HF) or low fertility (LF) bull.

**FIGURE 4:**
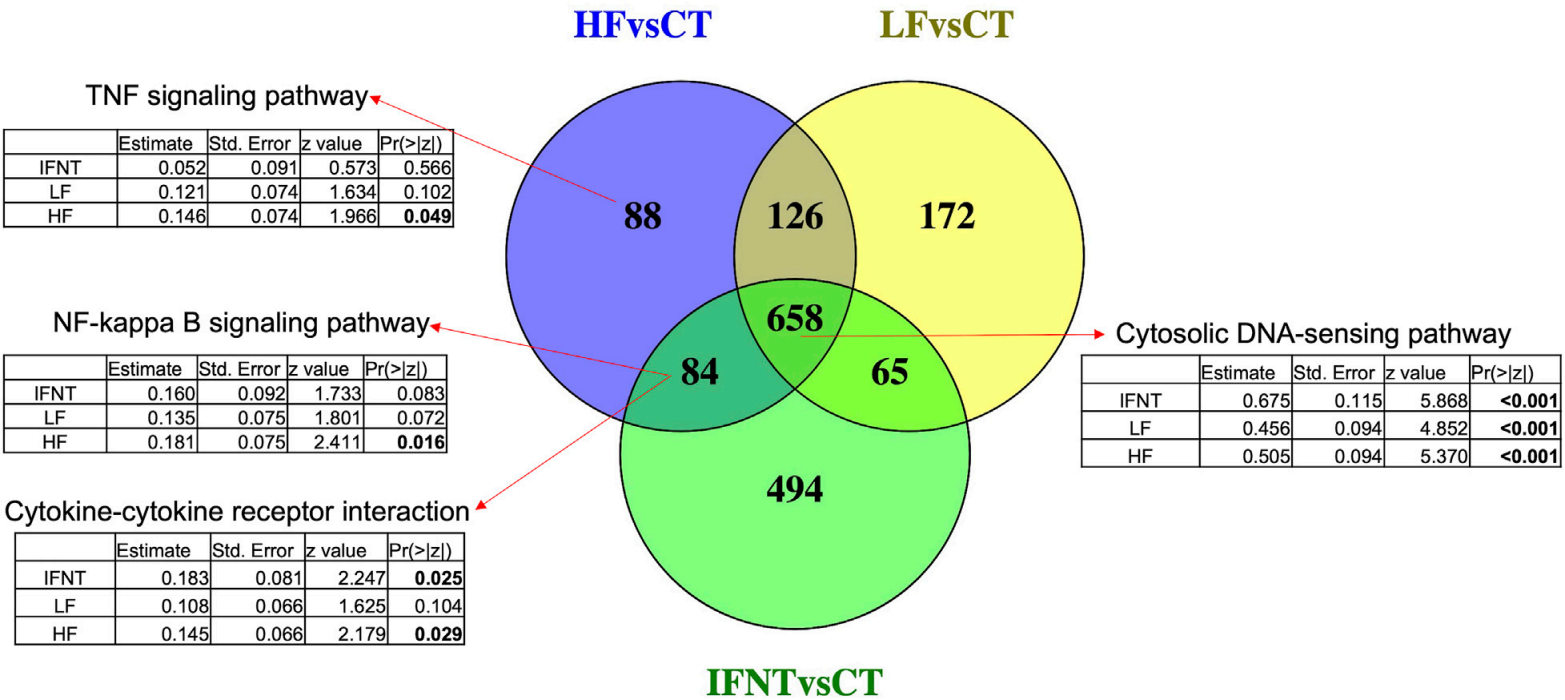
Venn Diagram of the differentially expressed genes between each group and the Control. Overlapping genes between the three comparisons enriched IFN-production pathways, such as cytosolic DNA-sensing. The other arrows indicate the enriched pathways by the corresponding genes. Below each pathway are shown the coefficients for the group effect in the expression of all genes in such pathway, modeled by negative binomial regression. Significant *p*-values are in bold. RNAseq data from endometrial explants exposed to nothing (Control, CT), interferon tau (IFNT), or a Day 15 conceptus from a high fertility (HF) or low fertility (LF) bull.

As expected, and based on comparison between groups, it was evident that the transcriptome of Control (unstimulated) samples was very distinct from the other groups (Supplementary Figure S1C). The numbers of DEG (FDR<0.05) were: IFNT vs. Control: 1301; HF vs. Control: 956; LF vs. Control: 1021; and HF vs. LF: 2 (Supplementary Table S1). The majority of DEG induced by IFNT or conceptuses were upregulated compared to untreated Control samples (Figure 3). When the DEG were compared in a Venn Diagram, unsurprisingly, the majority of DEG (658) were common to all comparisons (Figure 4). Functional analysis showed that these genes were associated with pathways related to IFNT production, such as viral diseases, antigen presentation, and cytosolic DNA-sensing pathways. The 88 non-overlapping genes between HF vs. CT enriched the TNF signaling pathway, while the overlapping 84 genes between HF vs. CT and IFNT vs. CT enriched the cytokine-cytokine receptor interaction, chemokine and NF-kappa B signaling pathways. The remainder of the overlapping and non-overlapping genes did not enrich any pathway at FDR<0.05.

Looking at the effect of each group on the expression of all the genes in the mentioned pathways, according to a negative binomial regression model, it was evident that the three groups strongly affected genes related with IFNT production, such as cytosolic DNA sensing pathway. Both IFNT and HF groups induced genes involved in cytokine-cytokine receptor interaction, while genes in the TNF and NF-Kappa B signaling pathways were modified only by the HF group.

### 3.2 Endometrial genes responding to conceptuses derived from HF or LF bulls

#### 3.2.1 Differentially expressed genes

**FIGURE 5:**
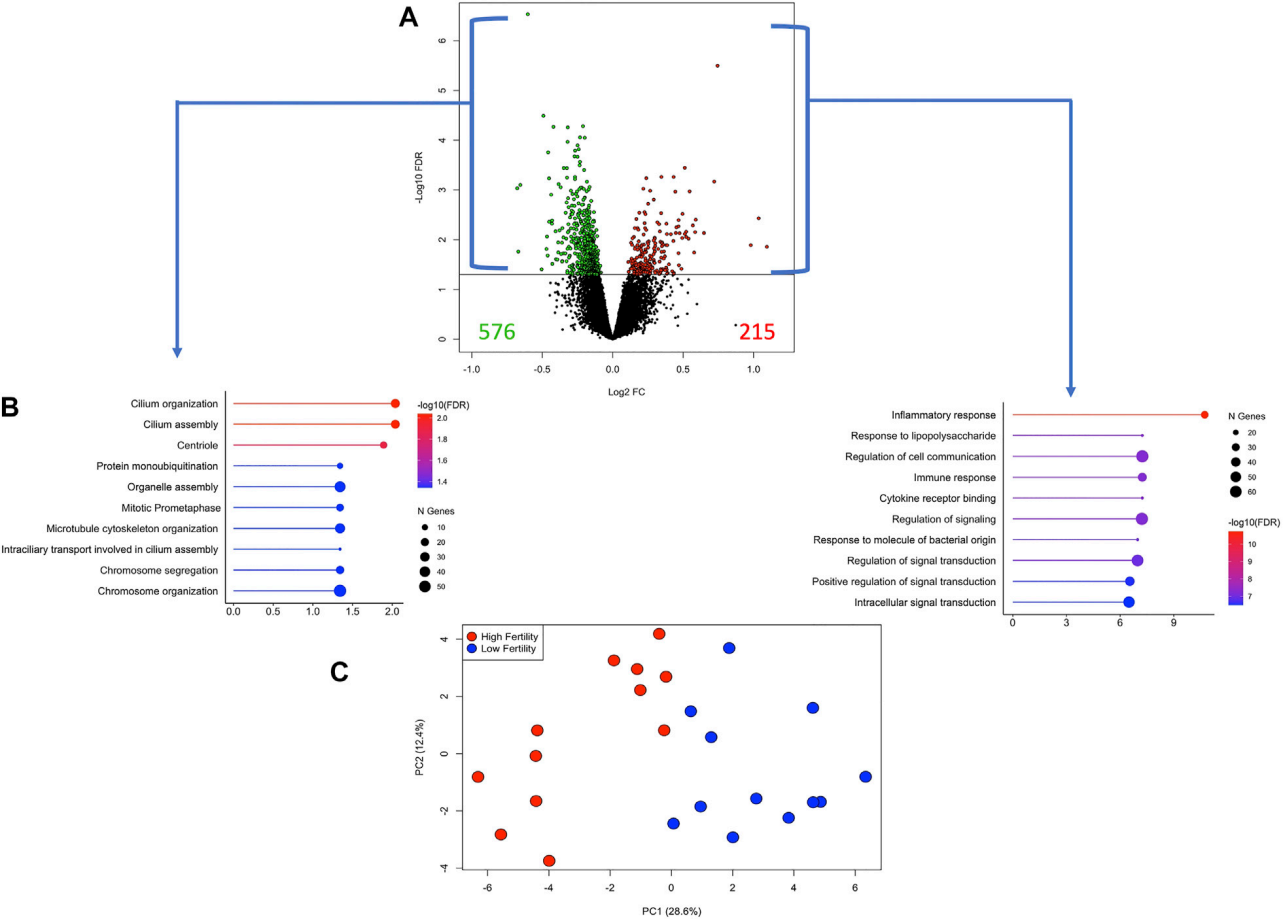
Differentially expressed genes (DEG) in endometrial explants exposed to a Day 15 conceptus from a high fertility (HF) or low fertility (LF) bull. **(A)** The volcano plot shows the number of DEG more expressed in the HF or LF groups as red or green dots, respectively. The horizontal black line was drawn at *p*-value = 0.05. **(B)** Lollipop plots representing the enrichment analysis from DEG more expressed in HF (right) or LF (left) groups. The length of the line (and its colour) indicates the fold change difference while the size of the dot indicates the number of genes involved. **(C)** Principal component analysis plot constructed with the expression of the DEG. Samples belonging to HF or LF groups are colored in red or blue, respectively.

The differences between the HF and LF groups were subtle, as can be observed in Supplementary Figure S1C. Therefore, DEG were selected based on the unadjusted *p*-value <0.05. While there is a risk with this exploratory approach of naming false positive DEG, we used two different approaches (WGCNA, sPLS-DA) to validate the findings. There were 791 DEG, of which 215 and 576 genes were more expressed in the HF and LF groups, respectively (Figure 5 and Supplementary Table S1). Enrichment analysis from DEG more expressed in HF revealed pathways associated with inflammatory and immune response, cytokine-receptor binding and regulation of signalling while in the LF group enriched pathways were associated with cilium organization and assembly and cell cycle parameters (Figure 5B). Using the expression of these genes in a PCA, a narrow separation of both groups of samples in the first component was evident (Figure 5C). Functional analysis showed that genes induced by conceptuses in the HF group were strongly related to immune function, enriching 30 immune-response related pathways (Supplementary Table S2). TNF and NF-Kappa B signalling were the top affected pathways. Genes stimulated by conceptuses in the LF group enriched only four pathways: Ubiquitin mediated proteolysis, cell cycle, oocyte meiosis and ribosome biogenesis in eukaryotes.

#### 3.2.2 Co-expressed genes

**FIGURE 6:**
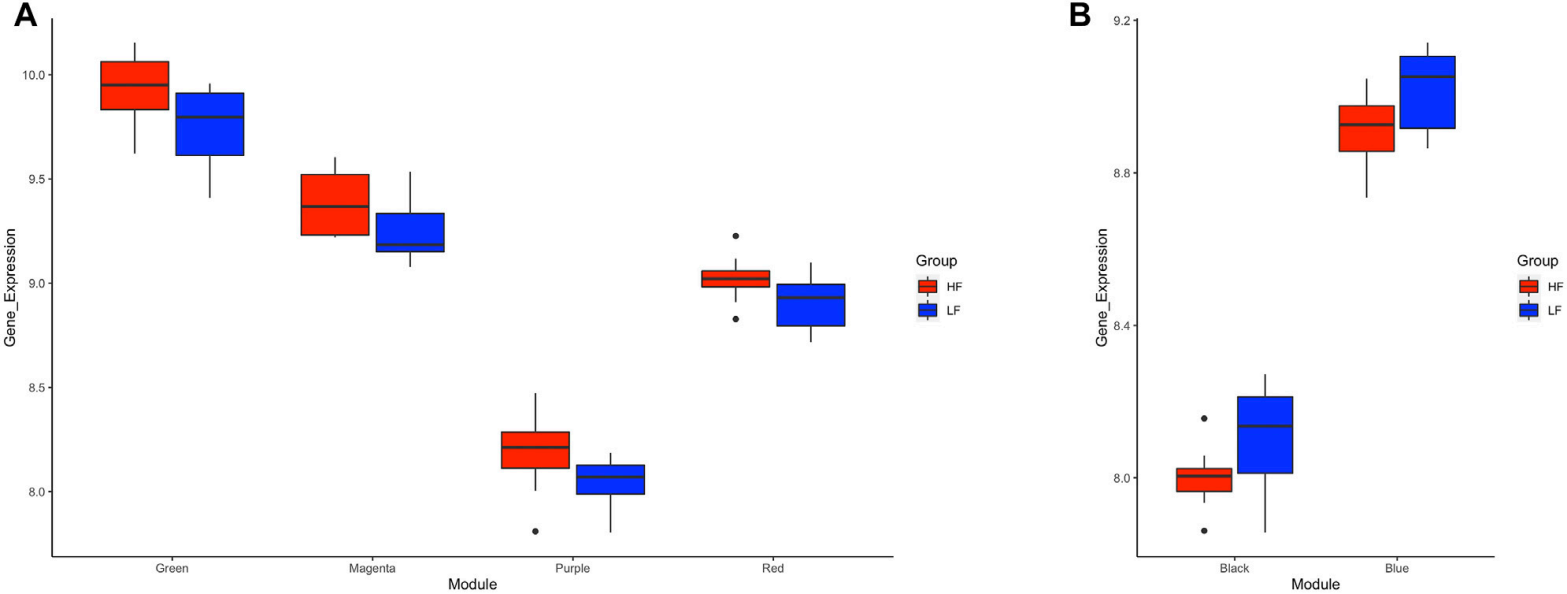
Box plots of average transformed expression for genes in modules correlated with high fertility (HF) or low fertility (LF) groups. Genes in each module are co-expressed, according to weighted gene co-expression network analysis (WGCNA). Modules correlated (*p* < 0.05) with **(A)** HF group or **(B)** LF group. RNAseq data from endometrial explants exposed to a Day 15 conceptus from a HF or LF bull.

Application of the WGCNA procedure identified four (purple, green, magenta and red) and two (blue and black) modules or clusters correlated with the HF group or LF group, respectively (Supplementary Figure S2 and Supplementary Table S3). Genes in such modules showed higher expression in the correlated group than in the other group (Figure 6) and were subjected to functional analysis. Genes in both the purple and green modules were involved in immune-related pathways. Again, TNF and NF-Kappa B signalling pathways were the top de-regulated pathways by the genes in the green module. Genes in the magenta and red modules were not enriching any pathway. For the modules correlated with the LF group, genes in the blue module enriched ribosome biogenesis in eukaryotes, mRNA surveillance pathways, and ubiquitin-mediated proteolysis, while genes in the black module were involved in RNA transport and cell cycle (Supplementary Table S4).

**FIGURE 7:**
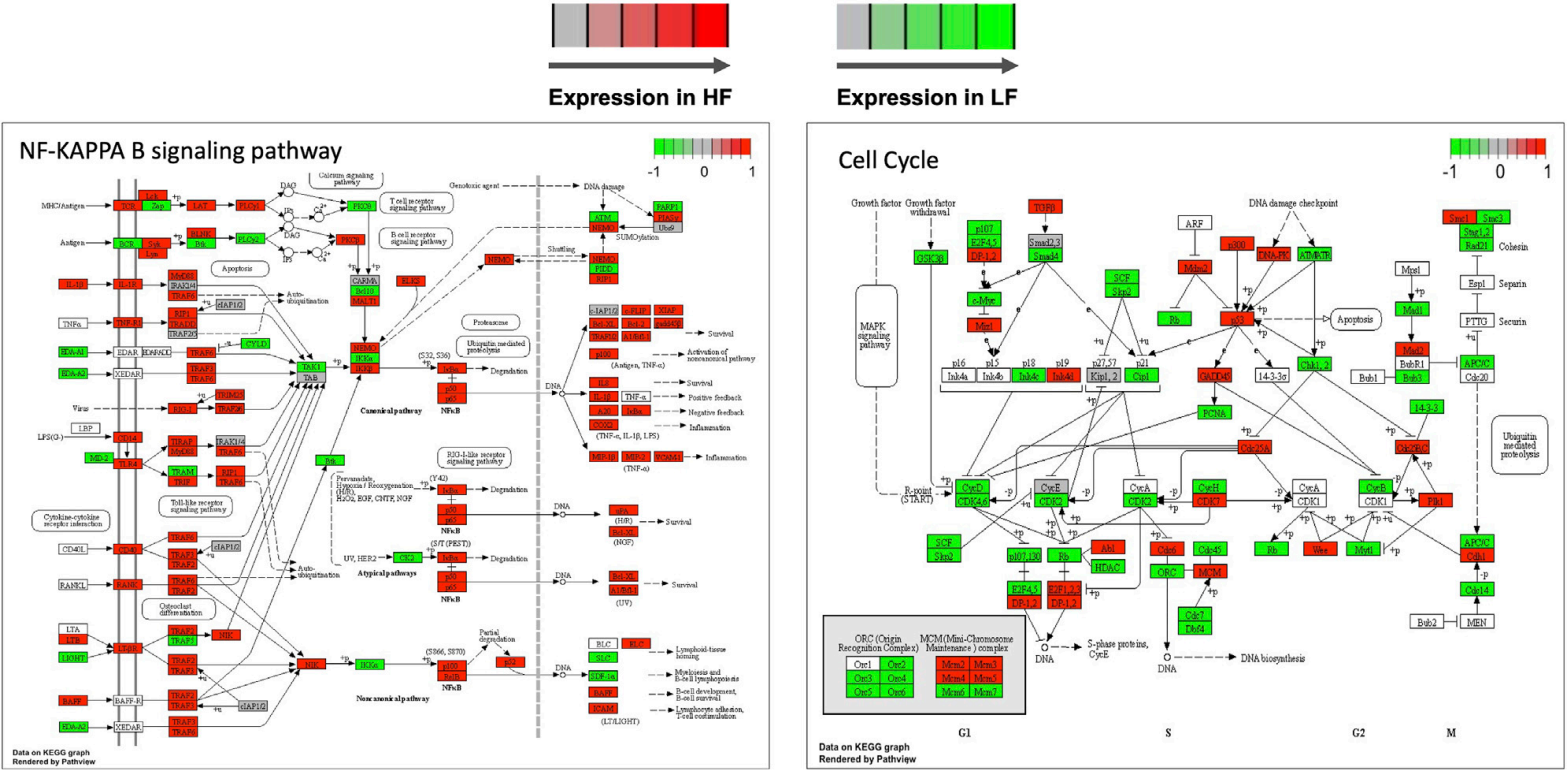
Selected KEGG pathways mapped with the expression of genes in high fertility (HF) or low fertility (LF) groups. The more intense the red or green each gene is colored, the more expressed is the gene in the HF or LF groups, respectively. RNAseq data from endometrial explants exposed to a Day 15 conceptus from a HF or LF bull.

Genes in the TNF and NF-Kappa B signalling, cell cycle, and ribosome biogenesis in eukaryotes pathways were mapped according to their expression in HF and LF groups. As expected, most of the genes in the first two pathways were more expressed in the HF group while the opposite occurred with the last two pathways, confirming the findings of the functional analysis for the correlated modules. Examples of two of these pathways are shown in Figure 7.

#### 3.2.3 Discriminative genes

**FIGURE 8:**
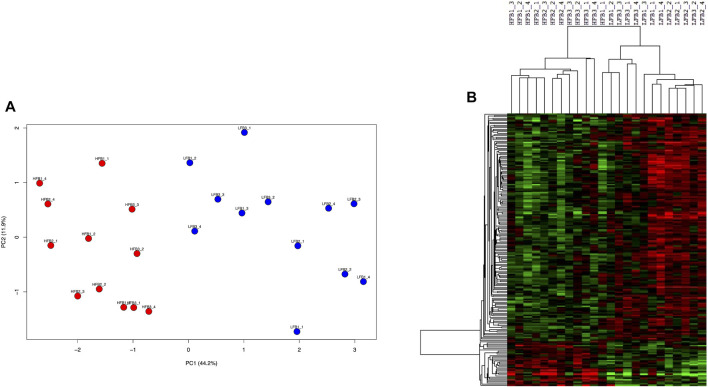
Discriminative endometrial genes for high fertility (HF) or low fertility (LF) groups selected by a supervised analysis. The 200 genes were identified through sparse-partial least square-discriminant analysis. **(A)** Principal component analysis of the HF and LF samples plotted according to the gene expression. **(B)** Hierarchical clustering and heat map of the selected genes. RNAseq data from endometrial explants exposed to a Day 15 conceptus from a HF or LF bull.

Selection of genes through the supervised sPLS-DA method resulted in 200 genes (Supplementary Table S5) which clearly separated both HF and LF groups in the first component in a PCA plot (Figure 8A). Almost all (199 of 200) of these genes were significant when HF was compared to LF in the DEG analysis (*p*-value<0.05). Most of them (167) were more expressed in the LF group, while the remaining 33 genes were up-regulated in the HF group (Figure 8B). These 33 genes enriched B cell receptor and adipocytokine signalling pathways (*p*-value <0.05), which included two NF-Kappa B-related genes (NFKB inhibitor epsilon and RELA proto-oncogene, NF-kB subunit). On the other hand, the 167 genes more expressed in the LF group were in nucleotide excision repair, spliceosome, cell cycle, and ubiquitin mediated proteolysis pathways (*p*-value <0.05).

### 3.3 Most relevant endometrial genes responding to conceptuses derived from HF or LF bulls

Genes selected after the application of three methods (differential expression analysis, WGCNA and sPLS-DA) detailed above were employed to predict the group (HF or LF) according to their expression. As expected, the 200 genes identified through sPLS-DA showed the best performance, since 90% of the times the group prediction was correct, and only one sample was misclassified once. The 791 DEG correctly predicted the groups on eight occasions, while in the remaining two occasions the accuracy was 83% and 90%. Finally, the co-expressed genes in the purple, green, black and blue modules (2198 in total), determined by WGCNA, correctly predicted the group twice, while the prediction accuracy ranged from 78 to 88% in the remaining evaluations (Supplementary Table S6).

**FIGURE 9:**
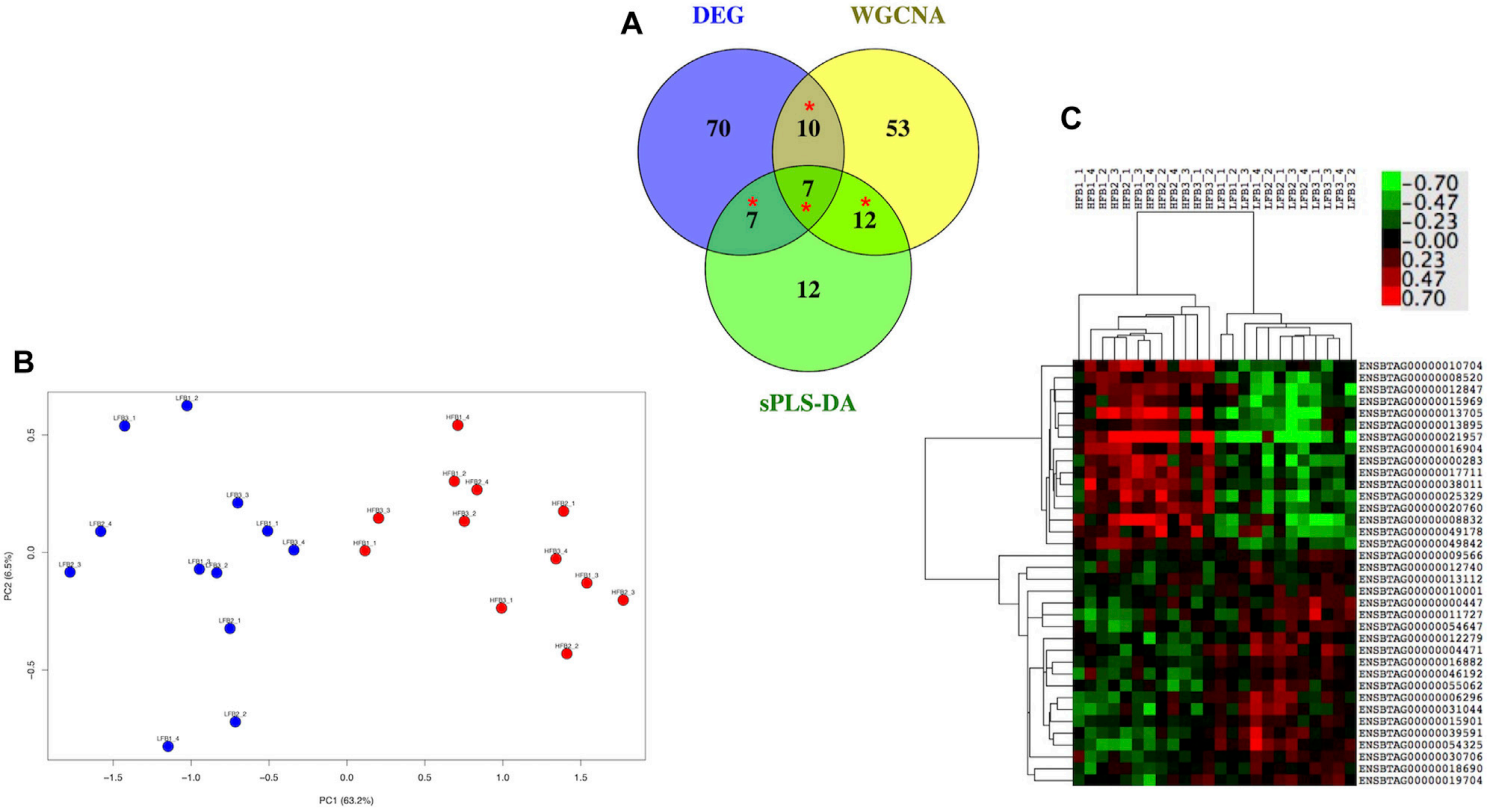
Most relevant genes related to high fertility (HF) or low fertility (LF) groups determined though a machine learning approach. Genes with at least 50% of AUC to predict the group were retained from the genes selected by three methods (DEG, WGCNA, sPLS-DA; see Figure 1). **(A)** genes were compared in a Venn Diagram to identify 36 most relevant genes (*). **(B)** Principal component analysis of the HF and LF samples plotted according to the expression of the 36 genes; **(C)** Hierarchical clustering and heat map of the 36 genes. RNAseq data from endometrial explants exposed to a Day 15 conceptus from a HF or LF bull.

There were 94, 82 and 38 genes with a least 50% of AUC in all the 10 evaluations for DEG, WGCNA and sPLS-DA, representing 11.9%, 3.7%, and 19% of the total number of inputted genes, respectively. When these genes were compared in a Venn Diagram, 36 genes were shared in at least two of the methods (Figure 9A). The list of the 36 genes is shown in Supplementary Table S7. These genes clearly distinguished the HF from the LF bulls in a PCA plot and hierarchical clustering (Figures 9B,C); 16 and 20 of them were more expressed in the HF and LF group, respectively (Figure 9C). The ontological terms associated with each annotated gene are described in Supplementary Table S8.

## 4 Discussion

The response of the bovine endometrial transcriptome to various stimuli has been characterized in detail by several groups during the past two decades. Such stimuli include progesterone (McNeill et al., 2006; Forde et al., 2009; Forde and Lonergan, 2012), pre-hatching embryos (Sponchiado et al., 2017; Talukder et al., 2017; Passaro et al., 2018) and post-hatching elongating conceptuses (Forde et al., 2011; Spencer, 2013; Mathew et al., 2019; Sánchez et al., 2019). However, the effect of sire fertility status on conceptus-induced changes in the transcriptome has not been investigated previously. Thus, the underlying factors that regulate or reflect communication between conceptuses derived from high and low fertility sires and the endometrium are unknown. The current study combined *in vitro* production of blastocysts, multiple ET and conceptus-endometrial explant co-culture to investigate the response of the endometrium to conceptuses derived from sires designated as either HF or LF status. In order to minimize the influence of host uterine environment on conceptus-induced response, embryos sired by bulls of varying fertility status were equally distributed across explants from the same heifers. The main findings from this study are: 1) conceptuses from HF sires stimulated pathways involved in immune response which LF conceptuses failed to stimulate, 2) DEGs with higher expression from HF-derived conceptuses were exclusively involved in TNF and NF-Kappa B signaling pathways.

Reproductive failure can be attributed to a multitude of factors affecting both the female and the male. In the female, these include compromised oocyte quality, leading to poor embryo quality and/or an inappropriate reproductive tract environment to support early embryo development, conceptus elongation, and implantation. The reduced ability of LF sires to establish pregnancy is multifactorial and involves factors such as sperm motility and fertilizing ability and any potential consequences of compromised quality on subsequent embryo development. The challenges in understanding the causes of poor fertility in monotocous species such as cattle (and humans) are quite different in males and females; on the female side, reproductive success tends to be “all or nothing”, in that, typically, a single oocyte is ovulated and the female has, thus, one chance to become pregnant during each estrous cycle. In contrast, an individual fertile sire has billions (natural mating) or millions (AI) of opportunities to get a female pregnant at each service event due to the high number of sperm deposited in the reproductive tract of the female, and even in circumstances where a proportion of those gametes are compromised (e.g., after freeze-thawing), sufficient viable sperm can remain to effectuate a successful fertilization. Therefore, while a male can have compromised fertility for a plethora of reasons, getting to the root cause of poor fertility in the male can be challenging. Nonetheless, fertilization occurs in approximately 90% of matings and dysregulation in subsequent embryogenesis accounts for the vast majority of reproductive failures. We want to explore the potential contribution of sire fertility status to this phenomenon.

Amongst all mammalian species, the confidence with which we can assign a fertility status to males is nowhere nearly as great as it is amongst bulls used in AI due to the high number of inseminations and subsequent pregnancy and birth records per bull, sometimes in the tens or even hundreds of thousands (Amann and DeJarnette, 2012). Despite rigorous assessments of sperm quality before semen is released, bulls used in AI still exhibit significant variation in field fertility. Numerous studies by our group and others have investigated aspects of sperm function in an attempt to explain divergent fertility in populations of AI bulls segregated based on pregnancy per AI (Sellem et al., 2015; Bernecic et al., 2021; Donnellan et al., 2022). Results tend to be rather inconsistent, probably a reflection of the multifactorial nature of sire subfertility as mentioned above and the fact that such AI bulls tend to be of high to medium fertility rather than being truly “low fertile” in terms of the extremes of fertility given that their semen has already undergone pre-freeze and post-thaw quality control checks.

Ortega et al. (2018) investigated the influence of Sire Conception Rate (SCR) on embryo development, conceptus elongation and pregnancy establishment in cattle. Low SCR bulls produced fewer Day 8 blastocysts after IVF, and more unfertilized oocytes and degenerated embryos in superovulated heifers. Day 16 conceptus recovery and length were not different between SCR groups and the conceptus transcriptome was not appreciably different between high and low SCR sires.

In a recent study from our group, using the same six bulls as used in the present study to generate elongated conceptuses for co-culture with endometrial explants, while differences in sire fertility were not reflected in fertilization rate, differences in embryo quality were apparent as early as Day 7 and likely contributed to a higher proportion of conceptuses surviving to Day 15 in HF bulls (O'Callaghan et al., 2021). In that study, following insemination of superovulated heifers, while overall recovery rate (total structures recovered/total corpora lutea) was 52.6% and was not different between groups, more embryos were at advanced stages of development, reflected in a greater mean embryo cell number on Day 7 for HF versus LF bulls. Furthermore, conceptus recovery rate on Day 15 following transfer of groups of Day 7 blastocysts produced *in vitro* using semen from the same HF (*n* = 3) and LF (*n* = 3) bulls to synchronized heifers was higher in HF (59.4%) versus LF (45.0%). Mean length of recovered conceptuses for HF bulls was not affected by fertility status (O'Callaghan et al., 2021). A representative number of these conceptuses, of similar length were used in this study for co-culture with endometrial explants.

As mentioned earlier, the transcriptomic response of the endometrium to the conceptus differs between those derived from AI vs. somatic cell nuclear transfer (Bauersachs et al., 2009; Mansouri-Attia et al., 2009), those derived from the transfer of *in vivo* vs. *in vitro* derived blastocysts (Mathew et al., 2019) and age-matched short and long conceptuses (Sánchez et al., 2019). We have extensively used the endometrial explant co-culture system to elucidate the crosstalk between the developing embryo and the maternal environment (Passaro et al., 2018; Mathew et al., 2019; Sánchez et al., 2019). Due to the maintenance of normal cellular and extracellular architecture in endometrial explants (Borges et al., 2012), some of the limitations of traditional cell culture can be overcome. For example, uterine explants allow the communication between resident populations of endometrial cells which cannot be achieved with current 2D and 3D *in vitro* cell culture technologies. We hypothesized that differences in endometrial response to conceptuses derived from HF and LF sires could be either dependent or independent of IFNT. We identified three categories of DEG compared to the Control endometrium: 1) those genes commonly responsive to exposure to IFNT and conceptuses, irrespective of sire fertility (n = 658); 2) those induced by the presence of a conceptus but independent of IFNT (*n* = 126), and 3) those specifically induced by conceptuses derived from HF (*n* = 88), or from LF (*n* = 172) bulls.

The first analysis involved identification of DEG between the three treatment groups (i.e., IFNT, conceptuses from HF bulls and conceptuses from LF bulls) compared to the Control. Consistent with our previous *in vivo* (Forde et al., 2015) and *in vitro* (Passaro et al., 2018; Mathew et al., 2019; Sánchez et al., 2019) studies, exposure of the endometrium to IFNT or a conceptus (from either a HF or LF bull) resulted in the upregulation of a large number of genes, not surprisingly mainly related to immune function. Between 950 and 1300 genes were altered due to treatment. While that, in itself, is not very informative, overlapping these DEG to highlight commonly differentially expressed genes, indicates that a large number (658) were shared by the 3 groups. Unsurprisingly given that one of the main factors secreted by the conceptus at this stage is IFNT, and consistent with our previous studies, functional analysis revealed that these genes were involved in biological processes related with IFNT production. These included classical interferon-stimulated genes (ISGs) such as *ISG15*, myxovirus resistance 1 (*MX1*), myxovirus resistance 2 (*MX2*), and 2′-5′-oligoadenylate synthase 1 (*OAS1*) amongst others. The 88 non-overlapping genes between HF vs. Control and the 84 between HF vs. Control and IFNT vs. Control are associated with immune response pathways such as TNF signaling, cytokine-cytokine receptor interaction, chemokine and NF-kappa B signaling pathways. These 88 and 84 genes were upregulated by HF conceptuses by inducing an immune response from the endometrium that did not occur with LF conceptuses. The 172 genes unique to LF conceptuses were mainly involved in cell cycle but did not significantly enrich any pathways.

Interestingly, a relatively large number of IFNT-independent genes were differentially expressed following exposure to a conceptus compared to the Control, consistent with our previous studies. Sánchez et al. (2019) found that 108 genes were induced exclusively by conceptuses and independent of IFNT, with 101 of these genes being exclusively induced by long and not by short age-matched conceptuses. Furthermore, conceptuses derived from AI altered 133 IFNT independent genes versus conceptuses produced by IVF which altered the expression of such 61 genes (Mathew et al., 2019). While the function of these genes, if any, in the process of maternal recognition of pregnancy is not known, we have previously reported the identification of proteins unique to the uterine lumen fluid of pregnant heifers and also produced by short-term *in vitro* culture of Day 16 conceptuses which could potentially be involved in facilitating the interactions between the conceptus and the endometrium during this period (Forde et al., 2015).

We then compared the DEGs only between the HF and LF groups—that is, those genes upregulated in the endometrium by a Day 15 conceptus from a HF vs. a LF bull. The differences between groups were more subtle than the previous analysis above. Nonetheless, enrichment analysis indicated that these DEG were strongly enriched for certain functions. For example, the HF group was enriched for immune response, response to stimuli and regulation of cell communication and signaling. These processes were not seen in the LF group, where the main processes related to cilium organization and organelle assembly, amongst others.

Prior to applying the adjusted *p*-value, there were 791 DEGs between HF and LF, with 215 and 576 genes more expressed in HF or LF, respectively (*p* < 0.05). DAVID software revealed that DEGs with higher expression in LF were involved only in cell cycle and proteolysis. However, upregulated DEGs by HF conceptuses were strongly associated with immune process pathways, such as TNF, NF-kappa B, cytokine-cytokine receptor interaction, and TLR signaling. These pathways were also enriched by upregulated DEGs by IFNT treatment compared to the Control. Furthermore, only the HF, and not the LF group, significantly affected the expression of most genes on these pathways (*p* < 0.05) according to a negative binomial regression model. While selecting DEG by raw *p*-value risks the calling of some false positives, the different approaches employed, including WGCNA validated the differences between HF and LF groups. WGCNA is potentially more insightful than differential expression analysis as it is used to reveal clusters of co-expressed genes which are expected to be functionally related. Also functional analysis revealed a strong enrichment of immune-related pathways, consistent with previous data.

As mentioned above, in cattle, fertilization occurs in approximately 90% of matings and issues in subsequent embryogenesis, some of which may be related to the sperm, account for the vast majority of reproductive failures. The differences in endometrial gene expression induced by Day 15 conceptuses from HF and LF sires are likely driven by genetic and/or epigenetic factors associated with the sperm from these sires. Recently, Diaz-Lundahl et al. (2021) assessed the gene expression of single, *in vivo* derived embryos from high and low fertility Norwegian Red bulls. There were 62 DEG between the groups and those more highly expressed in embryos derived from LF bulls were associated with increased active metabolism, a trait linked with lower embryo survival rates (Baumann et al., 2007; Leese et al., 2007). Embryos generated from high fertility sires displayed a higher expression of genes associated with anti-apoptosis and regulation of cytokine signalling and are involved in several functions in the pre and peri implantation period. Male fertility status-associated DNA methylation signatures in sperm can lead to altered transcriptomic profiles in bovine preimplantation embryos (Kropp et al., 2017). Furthermore, we have recently reported, using the same population of HF and LF bulls as used in the current study, that sperm DNA methylation patterns at discrete CpGs and genes involved in embryonic development are related to bull fertility (Stiavnicka et al., 2022).

In conclusion, consistent with our previous published reports, endometrial explants respond to a variety of stimuli in a manner similar to that observed *in vivo*. Culture of explants in the presence of IFNT or a conceptus, irrespective of the sire fertility status, upregulated a large number of, mostly immune-related, genes. Although differences in the endometrial response to conceptuses from HF vs. LF sires was more subtle, those from HF sires induced an immune response from the endometrium which was not observed in response to conceptuses derived from LF sires. These differences in the response of the endometrium to the conceptus at a critical point in pregnancy establishment (i.e., maternal recognition of pregnancy) may contribute to differences in fertility observed later during the first trimester of gestation.

## Data Availability

Original data are available at Gene Expression Omnibus (Accession GSE2054040).
