# Supplementary figures and images for: Influence of sire fertility status on conceptus-induced transcriptomic response of the bovine endometrium
Source: Front Cell Dev Biol. 2022 Aug 22;10:950443. doi: 10.3389/fcell.2022.950443 (PMC9442353; doi:10.3389/fcell.2022.950443)

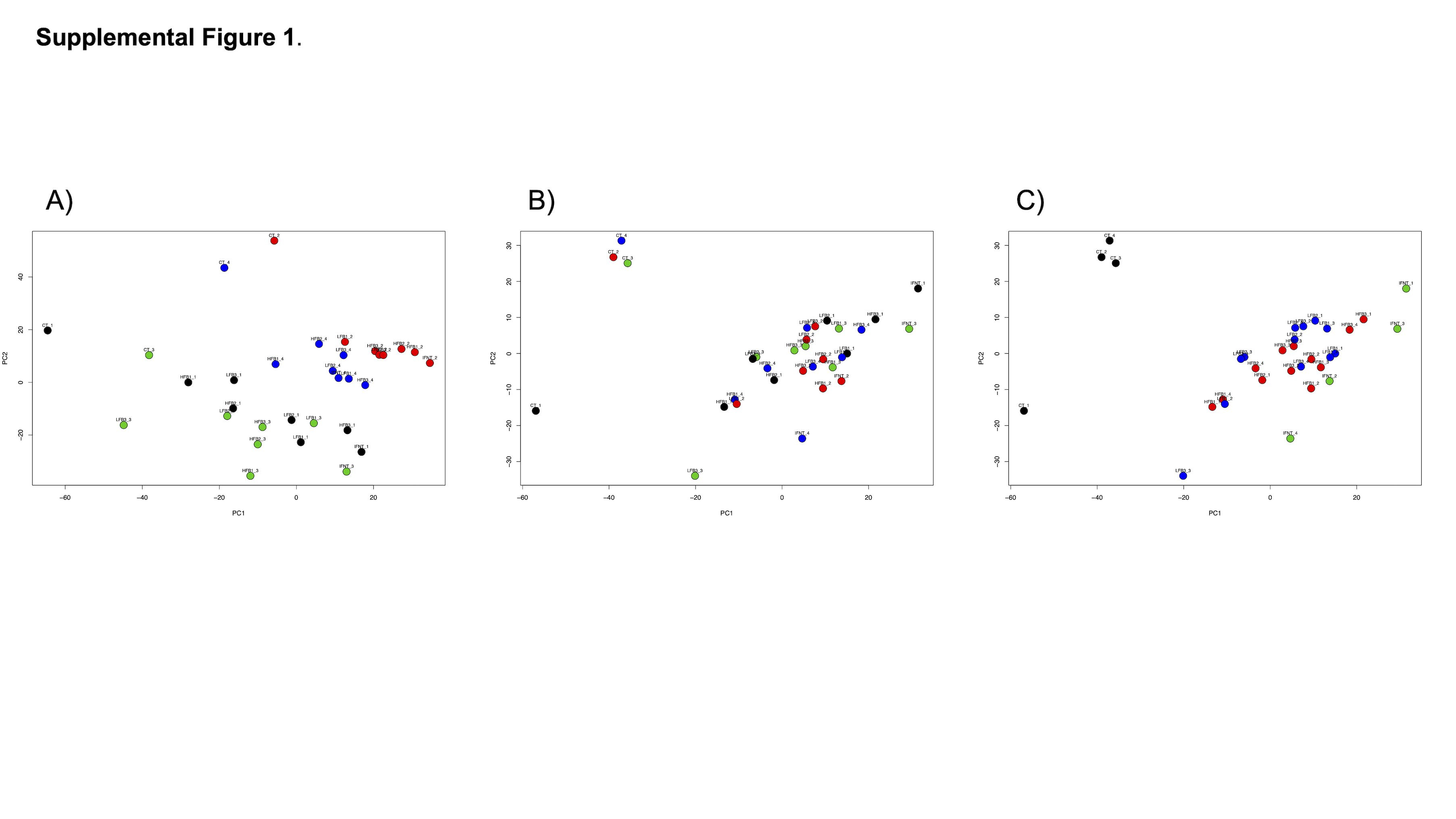

Supplement: Supplementary file 2 [file Image1.TIFF]

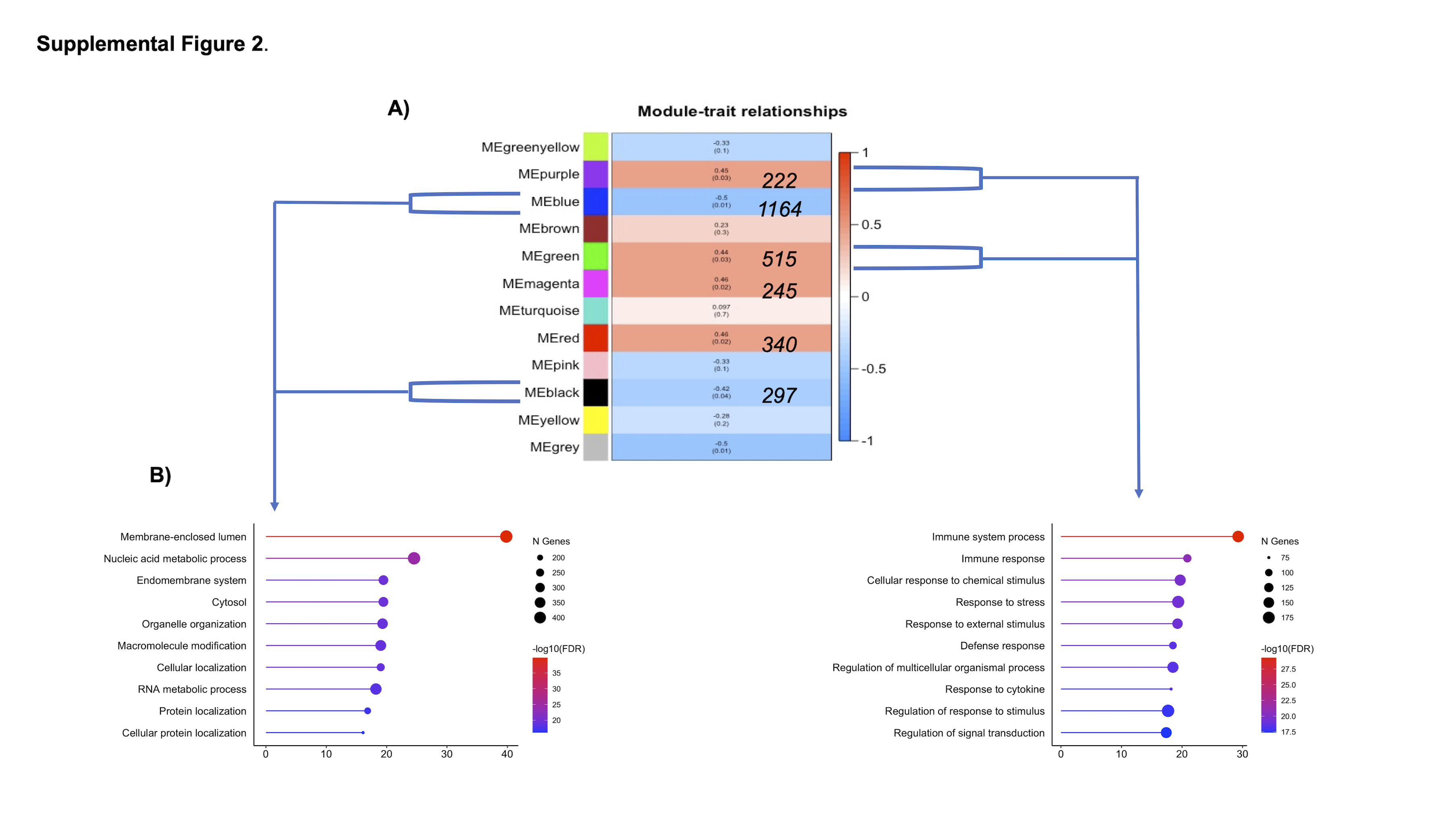

Supplement: Supplementary file 8 [file Image2.TIFF]
